# PfRON3 is an erythrocyte-binding protein and a potential blood-stage vaccine candidate antigen

**DOI:** 10.1186/1475-2875-13-490

**Published:** 2014-12-12

**Authors:** Xin Zhao, Zhiguang Chang, Zhiwei Tu, Shengchao Yu, Xiaoyan Wei, Jianhua Zhou, Huijun Lu, Ning Jiang, Qijun Chen

**Affiliations:** Key Laboratory of Zoonosis, Jilin University, Xi An Da Lu 5333, Changchun, 5333 China

## Abstract

**Background:**

Erythrocyte invasion by merozoites is an essential step in *Plasmodium falciparum* infection and leads to subsequent disease pathology. Proteins both on the merozoite surface and secreted from the apical organelles (micronemes, rhoptries and dense granules) mediate the invasion of erythrocytes; some of the molecules have been regarded as targets in the development of an anti-malaria vaccine. Recently, a subgroup of rhoptry neck proteins (PfRON2, PfRON4 and PfRON5) associated with the microneme protein apical membrane antigen AMA1 has been described as components of the moving junction complex that assists merozoite invasion into erythrocytes. However, unlike PfRON2, PfRON4 and PfRON5, the latest study suggested that PfRON3 might be located in the rhoptry bulb and participates in a novel PfRON complex (PfRON2, 3 and 4), but does not form a complex with AMA1. Additionally, the full-length PfRON3 protein possesses three transmembrane regions at the N-terminus, which is highly conserved among RON3 orthologues in the genus *Plasmodium*, *Toxoplasma gondii* and *Eimeria tenella*. Overall, these findings suggest that PfRON3 may play an important role in merozoite invasion into erythrocytes.

**Results:**

PfRON3 was primarily expressed during the late trophozoite stage, with a peak in transcription levels at 40 hours post-invasion. The subcellular localization of PfRON3 was confirmed that it is a merozoite rhoptry bulb protein. Additionally, the recombinant form of PfRON3 protein bound to the erythrocyte and was recognized by sera collected from malaria endemic areas in Africa, and anti-PfRON3 antibodies significantly inhibited merozoite invasion into erythrocytes.

**Methods:**

The expression of PfRON3 was analysed via real-time quantitative PCR, and the recombinant PfRON3 proteins were generated with an *Escherichia coli* expression system. The subcellular localization of PfRON3 was assessed with immunoelectron microscopy and immunofluorescence assay (IFA). The recognition PfRON3 by malaria immune sera was analysed with an enzyme-linked immunosorbent assay (ELISA). Erythrocyte-binding assays were performed using recombinant PfRON3 proteins and invasion inhibition assays were carried out with PfRON3-specific antibodies.

**Conclusion:**

This study confirmed that PfRON3 is a rhoptry protein with an erythrocyte-binding property, which is likely associated red blood cell invasion. PfRON3 is a potential vaccine candidate.

**Electronic supplementary material:**

The online version of this article (doi:10.1186/1475-2875-13-490) contains supplementary material, which is available to authorized users.

## Background

Among the malaria parasites that infect humans, *Plasmodium falciparum* is the most virulent parasite and was responsible for over 600,000 deaths in 2013 worldwide [1]. The pathogenesis of malaria occurs after the invasion of erythrocytes by merozoites and the replication of the obligate asexual intracellular parasites in the host erythrocytes. The erythrocyte invasion process begins with the attachment and proper re-orientation of the merozoite adhered to red blood cell surface via several merozoite surface proteins [2, 3]. Subsequently, an electron dense structure known as moving junction formed between the merozoite apical end and the erythrocyte membrane, involving rhoptry neck proteins and apical membrane antigen 1 (AMA1) [4]. Using the actin-myosin machinery, the merozoite pulls itself through the moving junction and eventually forms a parasitophorous vacuole inside the host erythrocyte [5]. During merozoite invasion into the erythrocyte, several rhoptry neck proteins are released and translocated onto the erythrocyte membrane acting as AMA1 receptors [6–8]. Meanwhile, AMA1 is secreted and inserted into the parasite plasma membrane to form the RON-AMA1 complex. Recently, three of the rhoptry neck proteins (PfRON 2, PfRON4 and 5) have been reported to be involved in the complex formation during invasion and bound to the micronemal protein apical membrane antigen PfAMA1 in *P. falciparum* [9–11]. In *Toxoplasma gondii*, four of the rhoptry neck proteins (TgRON2, TgRON4, TgRON5 and TgRON8) were found to associate with the moving junction and bind to TgAMA1 [12–14]. However, the interaction of *P. falciparum* RON proteins with AMA1 in the context of erythrocyte invasion remains further investigations.

A recent study has indicated that PfRON3 does not form complex with AMA1 but participates in a novel PfRON complex (PfRON2, PfRON3 and PfRON4). Its localization in the rhoptry body has been implicated [15], but its association with merozoite invasion is unclear. In this study, the expression of PfRON3 and its erythrocyte-binding activity was systematically investigated. It was found that PfRON3 bound to human red blood cell and specific antibodies to PfRON3 inhibited parasite invasion into erythrocytes.

## Methods

### Parasite culture

*Plasmodium falciparum* (strain 3D7) asexual stages were maintained in human O^+^ erythrocytes with 5% serum and 0.25% AlbuMAXII according to standard procedures [16]. The parasites were synchronized by three rounds of treatment with 5% sorbitol at 4 hours post-invasion and parasites at 8, 16, 24, 32, 40, and 48 hours post-infection were harvested.

### Transcription analysis of the PfRON3 gene via real-time quantitative PCR

Real-time quantitative PCR was carried out as previously described [17]. Briefly, RNA corresponding to six time points of post-invasion of the highly synchronized parasites was extracted with TRIzol Reagent (Invitrogen, Carlsbad, CA, USA) according to the manufacturer’s instructions. Following DNase treatment (TaKaRa, Dalian, China), cDNA was synthesized with AMV reverse transcriptase using an oligo(dT) primer. The following primers were used for quantitative RT-PCR: forward, 5-TTC GCT TCC TTC ATC GGT GC and reverse, 5-TCG TAA AAT TCG GTT GGG GC. Quantitative RT-PCR was performed using an ABI PRISM® 7500 Real-Time PCR System (Applied Biosystems) with SYBR® *Premix Ex Taq*^TM^ (TaKaRa). The *seryl-tRNA* synthetase gene (PF3D7_1205100) which is stably expressed during the erythrocytic stage of the parasite was selected as the internal control and used for normalization [18]. Analysis of the transcript levels relative to the average level of the internal control gene was calculated as 2^-ΔΔCt(PfRON3 gene)^ [19]. The experiment was repeated three times, and the mean and standard error were determined.

### Generation of PfRON3-specific antibodies

**Figure 1:**
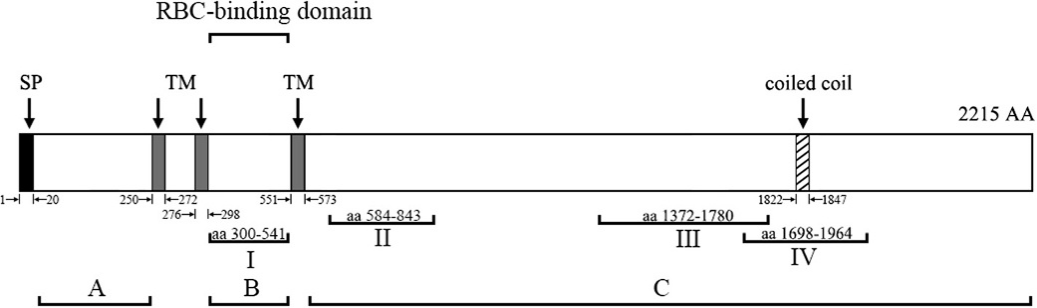
**Schematic map of the primary structure of PfRON3.** The PfRON3 protein contains 2215 amino acids with a calculated molecular mass of 263.15 kDa. A predicted signal peptide (SP, residues 1 to 20), three transmembrane regions (TM, residues 250 to 272, 276 to 298 and 551 to 573), and a coiled-coil region (aa 1822 to 1847) are indicated with dark, grey and slashed bars. The regions (I, II, III, and IV) corresponding to the four recombinant proteins generated are indicated underneath. The molecule is putatively divided into **A**, **B** and **C** regions based on the location of SP and TM regions (also see the Additional file 2).

For generation of PfRON3-specific antibody, HIS-tagged recombinant protein corresponding to region III of PfRON3 (Figure 1) was produced. The 1,224 bp fragment of the PfRON3 gene encoding a 408 amino acid fragment (region III, aa 1372–1780), was PCR-amplified from *P. falciparum* 3D7 strain blood–stage cDNA using the following primers: forward primer, 5-gga tcc TTT ACT AAT TTC TTA TTC TTA AGA AAC T and reverse primer, 5-ctc gag TTT TGT TCC ATA GTT TTC TGT ATT T. The amplicon was cloned into pET-32a (Novagen, Dusseldorf, Germany) vectors and expressed in *Escherichia coli* BL21 (DE3) [20]. The HIS-tagged recombinant PfRON3 protein (His-PfRON3-III) was purified using His GraviTrap columns (GE Healthcare) according to the manufacturer’s instructions. To generate anti-PfRON3-III sera, three female New Zealand white rabbits were immunized subcutaneously with 300 μg of purified His-PfRON3-III protein emulsified with Freund’s adjuvant. The immunizations were performed four times at two-week intervals, and the antisera were collected 14 days after the final immunization.

To generate specific antibodies to MSP-1-42, the gene fragment was amplified with primer MSP-1-42-F (5′- gaattc CCA CAA CTG AAG ATG GGG GTC AC-3′) and MSP-1-42-R (5′- ctcgag TGT AGA TGA TGT TCC AGT TA-3′). The gene fragment was cloned into pET-32a, and the recombinant protein (His-tagged PfMSP1-42) was expressed and purified as described above. Four female Wister rats respectively received four immunizations with 100 μg of purified His-tagged PfMSP1-42 protein emulsified with Freund’s adjuvant. The anti-PfMSP-1 antibodies were used as controls. Specific IgG fractions of the immunized rat and rabbit sera were affinity-purified with Protein G Sepharose^TM^ 4 Fast Flow (GE Healthcare) and Protein A Sepharose^TM^ 4 Fast Flow (GE Healthcare) according to the manufacturer’s protocols.

### Expression analysis of PfRON3 with SDS-PAGE and Western blot assays

Schizont-rich parasites were harvested after 1% saponin treatment. The parasite proteins were then solubilized in SDS-PAGE loading buffer (250 mM Tris, 1.92 M glycine, and 1% SDS), incubated at 98°C for 5 min and subjected to electrophoresis under reducing conditions in a 12% polyacrylamide gel. The proteins were transferred onto 0.2 μm nitrocellulose membranes (Bio-Rad, CA, USA) using a semi-dry blotting system (Bio-Rad, CA, USA). The membranes were blocked with 5% defatted milk (Sigma, St Louis, USA) for one hour and then incubated with anti-PfRON3 IgG (1:1,000 dilution). The membranes were further incubated with horseradish peroxidase conjugated secondary antibody (GE Healthcare) and visualized with Immobilon Western Chemiluminescent HRP Substrate (Millipore, Billerica, MA, USA) using a LAS 4000 mini luminescent image analyzer (GE Healthcare).

### Localization of PfRON3 with specific antibodies in immunofluorescence assay (IFA)

Thin smears of schizont stage *P. falciparum*-infected erythrocytes were fixed with methanol at -80°C for 5 min, washed with PBST (PBS containing 0.1% Triton X-100) at room temperature (RT) for 15 min, and then blocked with PBS containing 5% defatted milk at 37°C for one hour. The smears were then incubated with rabbit anti-PfRON3 antibody (1:50 dilution) and a control rat anti-MSP1-42 antibody at 37°C for one hour, followed by incubation with both Alexa Fluor 488-conjugated goat anti-rabbit IgG (Invitrogen) and Alexa Fluor 594-conjugated goat anti-rat IgG (Life) secondary antibody (1:1,000) at 37°C for one hour. The parasite nuclei were stained with DAPI (Roche, Basel, Switzerland) at ambient temperature for 5 min. High-resolution images were captured with a fluorescence microscope (Olympus, BX 53).

### Localization of PfRON3 with immunoelectronic microscopy

Immunoelectronic microscopy was carried out as previously described [15]. Briefly, parasites were fixed for 15 min on ice in a mixture of 1% paraformaldehyde and 0.1% glutaraldehyde in 0.1 M phosphate buffer (pH 7.4). The fixed specimens were washed, dehydrated and embedded in LR White resin (Fluka, 82882-1EA-F) as described previously [21, 22]. The ultra-thin sections were blocked in PBS containing 5% defatted milk. The grids were then incubated at 4°C overnight with rabbit anti-PfRON3 or control IgG. After washing, the grids were incubated at 37°C for one hour with goat anti-rabbit IgG conjugated to 5 nm gold particles (Sigma) with 1:40 dilutions. The grids were then rinsed with distilled water, dried and stained with uranyl acetate and lead citrate. The samples were examined using a transmission electronic microscope (Hitachi H-7650, Japan).

### RBC-binding activity of PfRON3

To investigate the erythrocyte-binding activity of PfRON3, GST-tagged PfRON3-I, II, III and IV (Figure 1), and GST (negative control) were expressed in *E. coli* BL21 [18] and purified on glutathione - sepharose. Primer pairs for region I are PfRON3-I-F (5′- gga tcc AAT CTC AGA CAA TTA TAT AGA A) and PfRON3-I-R (5′-ctcgag ACT GGA ATA TGG ATT ATT CAA TG). The primers for region II are PfRON3-II-F (5′-ggatcc GAA AGA TAT GGT GTT TTA AAA C) and PfRON3-II-R (5′-ctcgag GGT ACC TTG GTG ATA AGT TTG TT). The primers for region III are PfRON3-III-F (5′-ggatcc TTT ACT AAT TTC TTT TTA AGA AAC T) and PfRON3-III-R (5′-ctcgag TTT TGT TCC ATA GTT TTC TGT ATT T), and the primers for region IV are PfRON3-IV-F (5′-gaattc GAT CAA AGT ACA ACC GCT GATG) and PfRON3-IV-R (5′-ctcgag TGG TCC ACC ATA TGC TTT TTC AC). Next, 200 μl of human erythrocytes were washed three times and resuspended in 200 μl of PBS. Subsequently, 100 μg of recombinant proteins were mixed with the erythrocytes respectively, and incubated at RT for three hours. After incubation, the erythrocytes were washed with PBS three times. Eventually, the proteins that bound to erythrocytes were dissolved on a 10% SDS-PAGE gel and detected via Western blot using anti-GST IgG (Sungene Biotech, 1:5,000 dilution), which was used to characterize GST-tagged proteins.

To test the erythrocyte-binding activity of the native PfRON3, parasites at approximately 44 h post-invasion were harvested by centrifugation at 1500 rpm and washed 3 times with cold PBS. The parasites were lysed by sonication in the presence of a cocktail of protease inhibitors (Sigma). The solution was centrifuged 10 min at 12000 rpm. The supernatant was collected and incubated with 100 μl erythrocytes at 37°C for 1 hour. The cells were washed 3 times with PBS. The protein that bound to erythrocytes was analysed by SDS-PAGE followed by Western-blot using PfRON3 specific antibodies as described above.

### Immunorecognition of PfRON3 by human sera

The recognition of His-PfRON3 by human serum samples collected from malaria-endemic regions in Africa [23, 24] was examined. ELISA plates (Nunc, Rochester, NY, USA) were coated with the recombinant protein (5 μg/ml, 50 μl per well) at 4°C overnight. The plates were washed four times with PBS containing 0.05% Tween 20. The coated wells were then blocked with 3% BSA in PBS for one hour at 37°C and subsequently washed four times. The malaria-infected serum samples (n = 10) were added at 1:100, 1:200, 1:400 and 1:800 dilutions and incubated for one hour at 37°C. After washing four times, an alkaline phosphatase-conjugated goat anti-human secondary antibody (Sigma, St Louis, USA) was added at a 1:20,000 dilution and incubated at 37°C for one hour. Finally, a substrate solution containing pNPP [4-Nitrophenyl phosphate disodium salt hexahydrate] (Sigma, St Louis, USA), 9.7% diethanolamine (pH 9.8) and 0.1 M magnesium chloride was added to the wells. A human negative serum from a Chinese individual was used as a negative control and TBST solution was used as a blank control. The cutoff point of OD value for a positive sample was set to be at least two times higher than that of the negative sample at any dilution point. The plates were read using a Biotek 93 micro-ELISA auto-reader 808 at 405 nm. The experiment was repeated three times and the OD_405_ values were represented by the mean and standard error values of the three experiments.

### Invasion inhibition assay with PfRON3 antibodies

The invasion inhibition activity of PfRON-specific antibodies was tested *in vitro* as previously described [25]. Briefly, PfRON3 specific IgG was purified from rabbit antisera with Protein A-sepharose 4 Fast Flow (GE Healthcare) as described above. Synchronized *P. falciparum* cultures (ring stage) with 0.3% to 0.5% parasitaemia and 5% haematocrit were incubated with affinity-purified anti-PfRON3 IgG, anti-MSP1-42 IgG and healthy rabbit IgG (negative control). The antibody concentrations were 250, 150, 100, 50, and 10 μg/ml. The inhibitory activity of the antibodies on merozoite invasion was tested over two cycles of parasite replication. Parasitaemia was determined via flow cytometry using a FACS (BD FACS Aria cell sorter, CA, USA) and 50,000 cells were analysed in each experiment. The parasite nuclei were stained with ethidium bromide (EB) and tested in triplicates, the mean and standard errors were determined. The invasion inhibition efficiency of the control rabbit IgG was arbitrarily set as 0%, and the inhibition efficiencies of the PfRON3 specific as well as that of the MSP-1-42 specific antibodies were calculated by comparing the parasitemia in the culture with the control IgG.

## Results and discussion

### The PfRON3 gene is primarily transcribed at the late developmental stage of *P. falciparum*

One study early [15] indicated PfRON3 protein was expressed at late stage of *P. falciparum*. To determine the transcription of the PfRON3 gene during the blood-stage of *P. falciparum* 3D7 strain, real-time quantitative PCR analyses were performed by collecting highly synchronized parasite cultures at eight-hour intervals. Transcription of the PfRON3 gene was determined to start at approximately 24 hours and reached a peak at approximately 40 hours post-invasion (Additional file 1: Figure S1). The data further confirmed the earlier reports that the PfRON protein family is predominantly expressed at late schizont stage [10, 26].

### The purification of recombinant proteins

**Figure 2:**
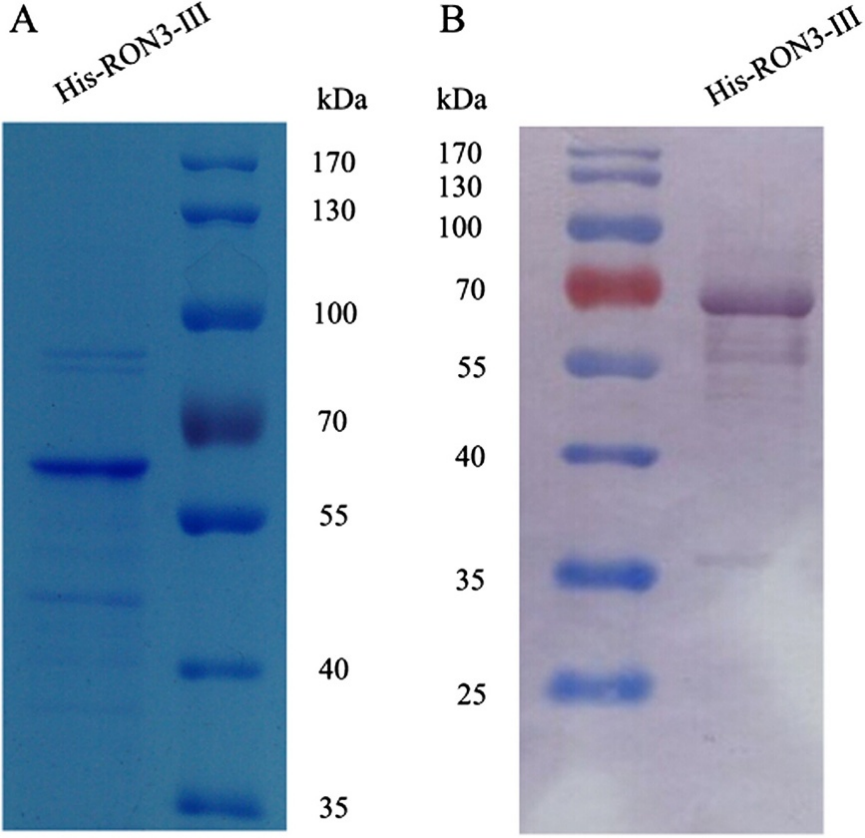
**Generation and purity of recombinant PfRON3 protein. (A)** SDS-PAGE analysis of purified His- PfRON3-III protein, which was generated using an *E. coli* expression system. The purified His-PfRON3-III (66.1 kDa) protein was resolved on a 12% SDS-PAGE gel and stained with Coomassie brilliant blue R-250. **(B)** Western blotting analysis of purified His-PfRON3-III protein under reducing conditions with anti-His-tag IgG. The smaller bands are degraded products of the full-length protein.

To further characterise the potential function of PfRON3, HIS-tagged recombinant protein (His-PfRON3-III) corresponding to the third region of the molecule (Figure 1) was expressed in *E. coli*, and purified via affinity chromatography on His GraviTrap columns. The quality of the purified recombinant protein was determined via 12% SDS–PAGE (Figure 2A) and Western blotting analyses (Figure 2B). A majority of the recombinant protein was in full-length, with only a small proportion of degraded fragments as seen in the Western-blot (Figure 2B). The recombinant proteins were soluble, which made it possible for the generation of functional antibodies and erythrocyte-binding assays.

### The expression and localization of PfRON3

**Figure 3:**
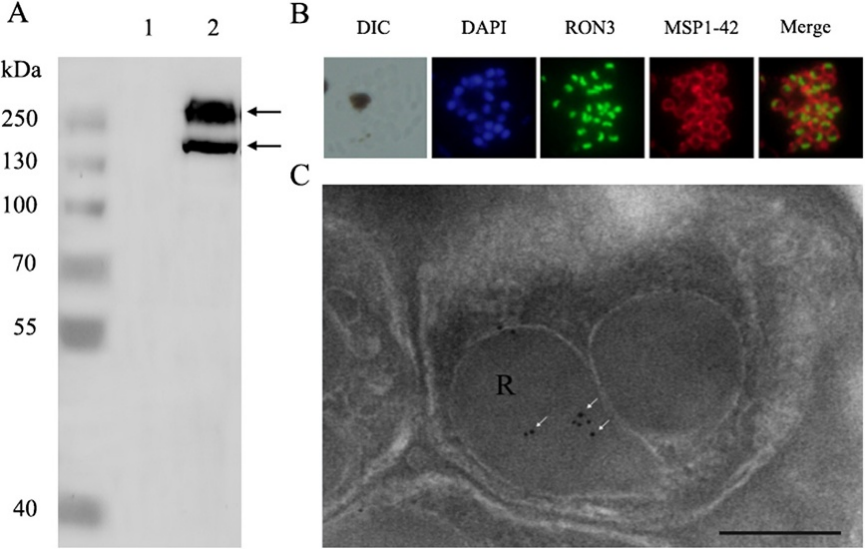
**The expression and localization of PfRON3 in** ***Plasmodium falciparum*** **merozoites. (A)** Detection of PfRON3 in schizonts. Lanes 1 and 2 represent healthy human red blood cells (negative control) and schizont-rich stage parasites. Two bands of approximately 260 and 180 kDa were detected in the schizont stage of the parasites (arrow-headed). **(B)** PfRON3 is expressed inside *Plasmodium* merozoites. Schizont stage parasites were dual-labeled with PfRON3-specific antibodies in combination with MSP1-42 antibodies. Nuclei are stained with DAPI. MSP1-42 protein was located at the merozoite membrane (red fluorescence) and RON3 was located inside merozoites (green fluorescence). **(C)** PfRON3 location shown via immunoelectronic microscopy. The section of merozoite in mature schizonts was labeled with purified rabbit anti-PfRON3 antibodies and subsequently detected with a secondary antibody conjugated with gold particles (arrow-headed). The black dots indicate signals from gold particles localized in the rhoptry body. Bar represents 200 nm.

The expression of PfRON3 protein was analysed via Western blot assay using an anti-PfRON3 antibody with protein extracts of synchronized schizont parasites. The antibody recognized a band larger than 250 kDa corresponding to the full-length PfRON3 protein (the molecular weight was estimated to be 263 kDa). A band smaller than 250 kDa was also detected using the anti-PfRON3 antibody (Figure 3A), which might represent the degraded or processed form of PfRON3 during the invasion of merozoites into erythrocytes. In the study of Ito *et al.* [15], the antibody primarily recognized the second band, which was different from ours. This could be due to the differences in the time points of parasites collected or the antibodies used in the two analyses. However, the data collectively indicated that PfRON3 was processed after expression.

To confirm the localization of PfRON3 in the merozoite, a dual label indirect immunofluorescence assay was performed using a rabbit anti-PfRON3 antibody with a rat anti-MSP1-42 (membrane marker) antibody as a control. The IFA results showed that the location of PfRON3 (green fluorescence) was distinct from that of MSP1-42 (red fluorescence), which has been previously confirmed as a merozoite surface protein (Figure 3B). The IFA data showed that the localization of PfRON3 protein was indeed distinct from membrane proteins and primarily located inside merozoites.

A previous report indicated that PfRON3 may be located in the rhoptry body [15]. In this study, the location of PfRON3 was re-analysed with immunoEM. Parasite sections in late schizont stages were incubated with RON3-specific IgG and subsequently with a secondary antibody labelled with gold particles. Gold particle signals were clearly detected in the bulb portion of the rhoptries (Figure 3C). The data further confirmed that PfRON3 is localized in the rhoptry bulb rather than the rhoptry neck.

### RON3 binds to human erythrocytes

**Figure 4:**
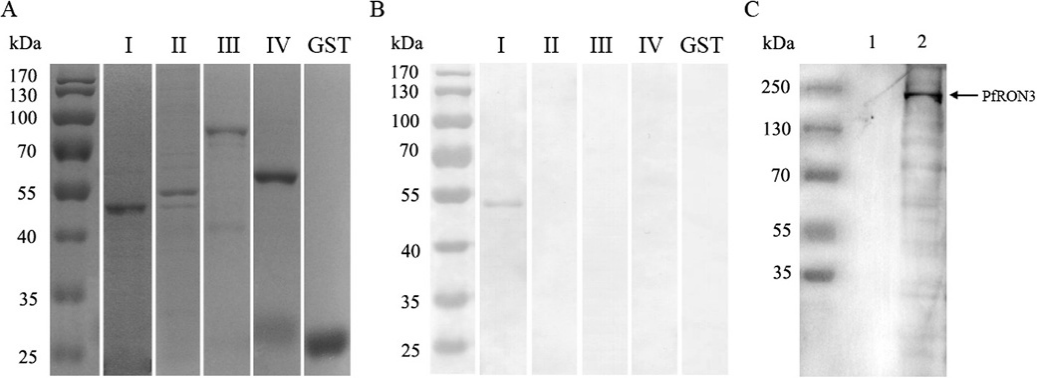
**Erythrocyte-binding activity of PfRON3 proteins. (A)** SDS-PAGE analysis of purified GST-tagged PfRON3 proteins. The purified RON3-I (52 kDa), RON2-II (54 kDa), RON3-III (74.1 kDa), RON3-IV (55.7 kDa) and GST (26 kDa) proteins were resolved in 12% SDS-PAGE gels and stained with Coomassie brilliant blue R-250 are shown. **(B)** Western blot analysis of the erythrocyte-binding activity of the recombinant GST-tagged PfRON3 proteins. The proteins were mixed with human erythrocytes separately and the binding was subsequently detected with a GST-specific mAb. The results show that only the recombinant PfRON3-I protein could bind to human erythrocytes. **(C)** Detection of the binding of the native PfRNO3 with human red blood cells. Soluble parasite proteins were incubated with human RBC and the binding of PfRON3 with RBC was detected with a specific antibody in Western-blot. The antibody only recognized the protein (PfRON3) (lane 2), but not reacted with any protein of RBC (lane 1).

Recombinant GST-tagged PfRON3-I, II, III, IV and GST proteins were tested for erythrocyte-binding activity. Results (Figures 1 and 4) showed that only the GST-tagged PfRON3-I protein, which corresponded the region between the second and third TM of PfRON3, was found to bind erythrocytes but not the other three regions. GST did not show any binding to erythrocytes either (Figure 4B). The erythrocyte-binding activity of PfRON3 was further confirmed with an assay where the native PfRON3 was found to bind human erythrocytes *in vitro* (Figure 4C). The data suggested that PfRON3 contains a region that mediates the interaction between merozoites and human erythrocytes. Further, the expression analyses in this study and that reported by Ito *et al.* [15], two forms of PfRON3 were identified in the parasite, one was likely the full-length PfRON3, and the other was around ≈ 80 kDa smaller than the whole molecule. Here, it was found that only the smaller version (≈180 kDa) of the molecule bound to erythrocytes, but not the whole molecule. This indicated that PfRON3 was most likely protelytically processed before it was released from the rhoptry organelle (Additional file 2: Figure S2). Only the smaller form of the molecule binds red blood cell. Additionally, previous studies suggested that PfRON2 and PfRON5 could bind to the RBC membrane and thereby served as a parasite-specific receptor of the AMA1 complex during invasion [27, 28]. The data of this study not only confirmed the reported by Zhang *et al*. [29], but also suggested that PfRON3 is a parasite ligand in the early phase of erythrocyte invasion. Thus, a majority of the PfRON proteins are erythrocyte-binding proteins participating in the interaction of the invading merozoite and erythrocytes.

### PfRON3 was recognized by the antibodies of individuals living in malaria-endemic regions and anti-PfRON3 antibodies inhibited merozoite invasion

**Figure 5:**
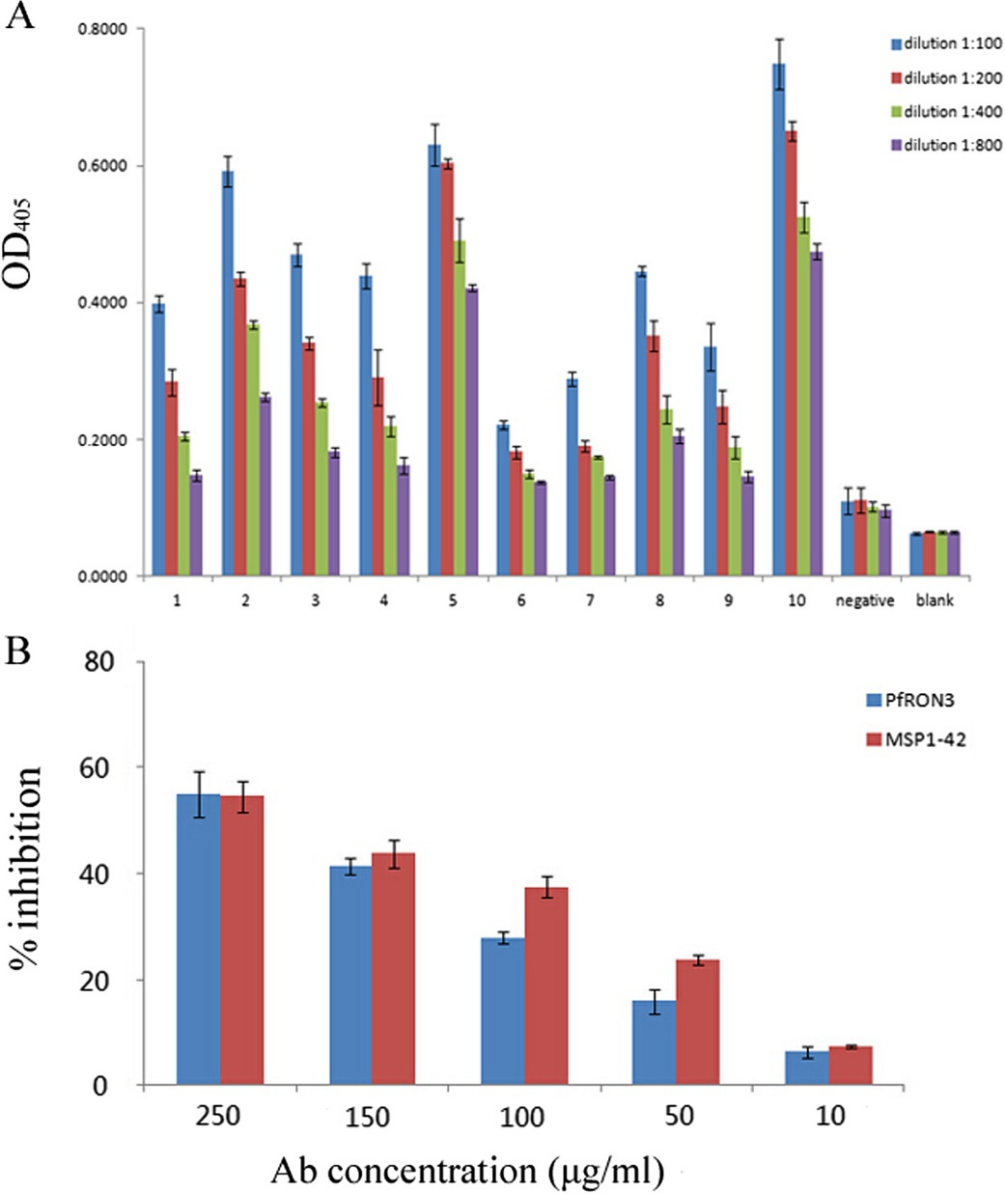
**Immunogenicity of PfRON3 and invasion inhibition of PfRON3-specific antibodies. (A)** Immunorecognition of PfRON3 by the sera of individuals living a malaria endemic area was analyzed via ELISA assay. The sera were diluted at 1:100 to 1:800. The results show that PfRON3 is well recognized by the antibodies in the sera, while the negative control sera did not react with the protein. The bars represent the mean and standard error values of the three experiments. **(B)** The inhibitory effect of PfRON3-specific IgG on merozoite invasion was tested. Anti-PfRON3 and anti-MSP1-42 specific IgG were diluted in concentrations ranging from 10 to 250 μg/ml. IgG from healthy rabbits was used as a negative control. The results show that PfRON3- and MSP-1-specific IgG inhibited parasite proliferation in a dose-dependent manner. Meanwhile, the control rabbit IgG had no inhibitory effect at any concentrations. The bars represent the mean and standard error values of the three experiments.

Invasion into human erythrocytes is the first step for the establishment of infection and successful transmission of the malaria parasites. Thus immune responses to parasite ligands participating in the invasion process will provide the first line immunity against malaria disease. Here the importance of PfRON3 as an immunogen during parasite infection was investigated in two approaches. First, the recombinant PfRON3 was tested for the recognition by the antibodies in the malaria immune individuals, and it was found that PfRON3 was well recognized by the sera of individuals living in the malaria endemic region (Figure 5A). Secondly, the antibodies to PfRON3 were found to sufficiently inhibit merozoite invasion in a similar manner as anti-MSP-1 antibody (Figure 5B). The data collectively indicated that PfRON3 is a molecule playing an important role in the process of erythrocyte invasion and immune responses to this antigen would likely provide certain immunity against severe malaria. Considering the importance of the PfRON complex in the merozoite invasion of erythrocytes and its high sequence similarity between different parasite strains/isolates, PfRON3 should be further investigated as a potential vaccine candidate.

## Conclusions

The data of this study indicate that the *PfRON3* gene was primarily transcribed at the late schizont stage, and the encoded PfRON3 is mainly localized in the rhoptry bulb, which is likely to be released and bind erythrocyte upon the interaction of the anterior end of a merozoite with the surface of an erythrocyte. PfRON3 is recognized by the antibodies of individuals living in malaria endemic areas. Importantly, PfRON3-specifc antibodies inhibited the parasite invasion into erythrocytes, thereby suggesting that PfRON3 could be a potential malaria vaccine candidate.

## Electronic supplementary material

Additional file 1: **Transcription of PfRON3 gene in the** ***Plasmodium falciparum*** **3D7 clone at different developmental stages.** The transcription levels of the PfRON3 gene at 8, 16, 24, 32, 40 and 48 hours p.i. were detected by QPCR. The transcription level at 16 hour was the lowest; the fold changes presented in the figure were all relative to that of 16 hour. The values of the fold changes were calculated by 2^-ΔΔCt^. (TIFF 788 KB)

Additional file 2: **Putative model of the location of PfRON3 after merozoite release.** The N-terminal region (A, indicated with dashed line) of PfRON3 was likely protelytically processed. The region B, associated with RBC-binding during merozoite invasion, might be located outside the parasite or parasitophorous vacuole membrane (PVM), while the region C is located inside the merozoite or PMV. (JPEG 33 KB)
