# Supplementary figures and images for: PfRON3 is an erythrocyte-binding protein and a potential blood-stage vaccine candidate antigen
Source: Malar J. 2014 Dec 12;13:490. doi: 10.1186/1475-2875-13-490 (PMC4295329; doi:10.1186/1475-2875-13-490)

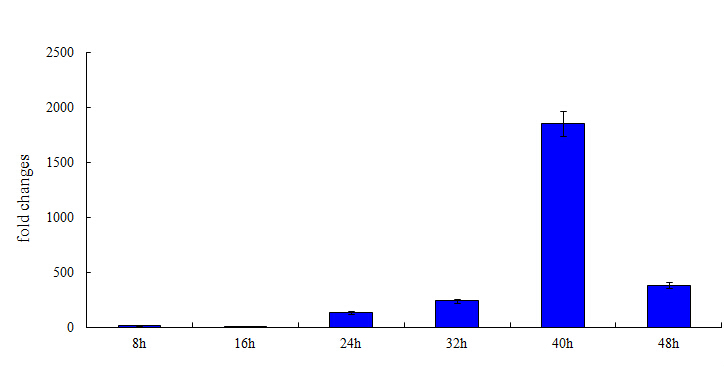

Supplement: Supplementary file 1 — Additional file 1: Transcription of PfRON3 gene in the Plasmodium falciparum 3D7 clone at different developmental stages. The transcription levels of the PfRON3 gene at 8, 16, 24, 32, 40 and 48 hours p.i. were detected by QPCR. The transcription level at 16 hour was the lowest; the fold changes presented in the figure were all relative to that of 16 hour. The values of the fold changes were calculated by 2-ΔΔCt. (TIFF 788 KB) [file 12936_2014_3634_MOESM1_ESM.tiff]

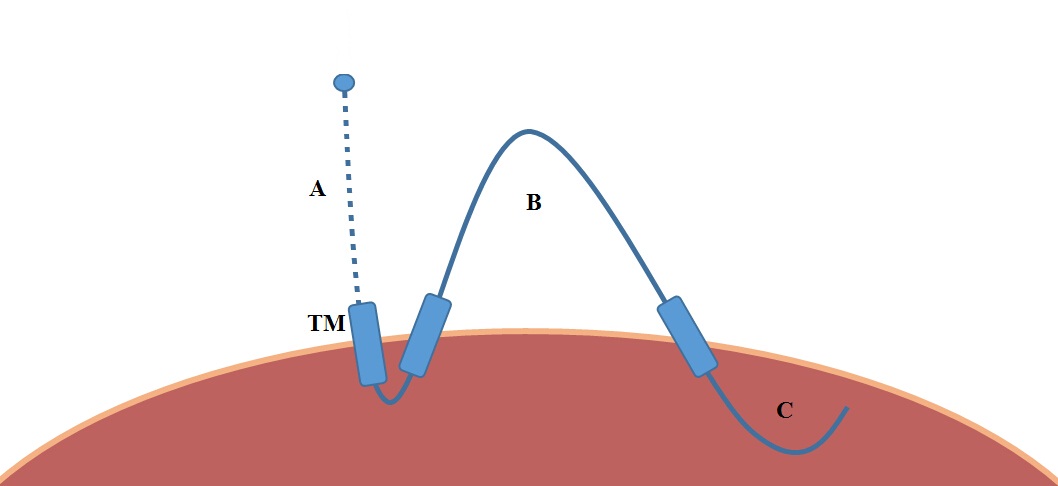

Supplement: Supplementary file 2 — Additional file 2: Putative model of the location of PfRON3 after merozoite release. The N-terminal region (A, indicated with dashed line) of PfRON3 was likely protelytically processed. The region B, associated with RBC-binding during merozoite invasion, might be located outside the parasite or parasitophorous vacuole membrane (PVM), while the region C is located inside the merozoite or PMV. (JPEG 33 KB) [file 12936_2014_3634_MOESM2_ESM.jpeg]
